# Current methods in structural proteomics and its applications in biological sciences

**DOI:** 10.1007/s13205-011-0037-1

**Published:** 2011-12-10

**Authors:** Babu A. Manjasetty, Konrad Büssow, Santosh Panjikar, Andrew P. Turnbull

**Affiliations:** 1 European Molecular Biology Laboratory, Grenoble Outstation and Unit of Virus Host-Cell Interactions, UJF-EMBL-CNRS, UMI 3265, 6 rue Jules Horowitz, BP181, 38042 Grenoble Cedex 9, France; 2 Department of Molecular Structural Biology, Helmholtz Centre for Infection Research, 38124 Braunschweig, Germany; 3 Australian Synchrotron, 800 Blackburn Road, Clayton, VIC 3168 Australia; 4 Cancer Research Technology Ltd., Birkbeck College, University of London, London, WC1E 7HX UK

**Keywords:** Protein structure analysis, X-ray crystallography, Bioinformatics, Structural proteomics

## Abstract

A broad working definition of structural proteomics (SP) is that it is the process of the high-throughput characterization of the three-dimensional structures of biological macromolecules. Recently, the process for protein structure determination has become highly automated and SP platforms have been established around the globe, utilizing X-ray crystallography as a tool. Although protein structures often provide clues about the biological function of a target, once the three-dimensional structures have been determined, bioinformatics and proteomics-driven strategies can be employed to derive their biological activities and physiological roles. This article reviews the current status of SP methods for the structure determination pipeline, including target selection, isolation, expression, purification, crystallization, diffraction data collection, structure solution, refinement and functional annotation.

## Introduction

One of the most spectacular recent achievements in life sciences has been the sequencing of the entire human genome, accomplished by the Human Genome Project. The resolution of the entire sequence of the human genome has resolved many unanswered questions relating to human life. The human body comprises a vast number of cells and each cell contains many thousands of different proteins necessary to maintain cellular function. Knowledge of the sequence of the human genome means that disease-associated abnormalities can now be detected at the genetic level. Furthermore, sequence comparisons can provide an insight into the evolutionary relationship between organisms. As of August 2011, the UniProtKB/Swiss-Prot database has contained in excess of half a million non-redundant sequence entries. Hence, it is clear that large-scale genomic projects have provided the sequence infrastructure for the in-depth analysis of proteins. A new fundamental concept of the proteome (PROTEin complement to a genOME) has emerged that aims to unravel the biochemical and physiological mechanisms of complex multivariate diseases at the functional and molecular level. As a consequence, the new science of proteomics has been established to complement physical genomic research. Proteomics can be defined as the *qualitative*and *quantitative*comparison of proteomes under different conditions, which aims to further characterize biological processes and functional protein networks (Naistat and Leblanc 2004; Petschnigg et al. 2011; Stults and Arnott 2005). However, the knowledge gleaned from the various genomes sequenced to date is not sufficient to understand the function of proteins within the cell. To characterize functional protein networks and their dynamic alteration during physiological and pathological processes, proteins have to be identified, sequenced, categorized and classified with respect to their function and interaction partners. To understand their functions at a molecular level, it is often necessary to determine their three-dimensional (3D) structures at atomic resolution.

During the past decade, the emerging field of structural proteomics (SP) has developed, representing an international effort aimed at the large-scale determination of the 3D structures of proteins encoded by the genomes of key organisms (Burley 2000; Joachimiak 2009; Manjasetty et al. 2007; Terwilliger 2011). Initiatives in SP research have led to the development of novel strategies and automated protein structure determination pipelines around the world (Table 1) (Chance et al. 2004; Manjasetty et al. 2008).

**Table 1:** Major centers for high-throughput structure determination around the world

No.	Country	Center	Web address	PDB entries
1.	Japan	RIKEN Structural Genomics/Proteomics Initiative	http://www.rsgi.riken.go.jp/	2,702
2.	USA	Midwest Center for Structural Genomics (MCSG)	http://mcsg.anl.gov/	1,389
3.	USA	Joint Center for Structural Genomics (JCSG)	http://www.jcsg.org/	1,234
4.	USA	New York Structural Genomics Research Consortium (NYSGRC)	http://www.nysgrc.org/	1,028
5.	Canada, UK, Sweden	Structural Genomics Consortium (SGC)	http://www.thesgc.org/	1,005
6.	USA	Northeast Structural Genomics Consortium (NESG)	http://www.nesg.org/	964

**Fig. 1:**
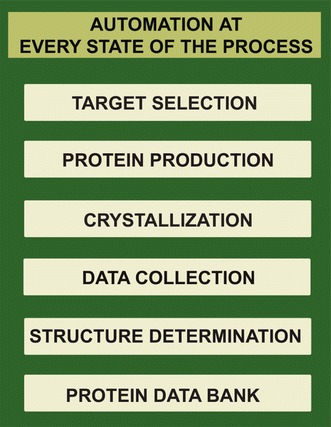
Process involved in SP using X-ray crystallography

**Fig. 2:**
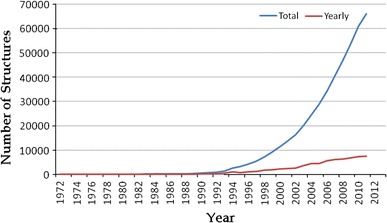
Exponential growth in the number of X-ray protein structures deposited in the Protein Data Bank

When protein structure analysis was first established in the late 1960s and the X-ray structures of myoglobin and hemoglobin were determined, the development of such a high-throughput (HT) infrastructure for protein structure analysis would have seemed like an impossible dream. The remarkable success and technological advancements since then have had a tremendous impact on throughput in protein structure determination and all stages of the pipeline have become more or less automated (Fig. 1). Currently, SP initiatives are generating protein structures at an unprecedented rate and have resulted in an exponential growth in the number of protein structures deposited in the Protein Data Bank (Fig. 2: 65979 PDB entries, as of August 2011). However, the number of solved protein structures in the PDB represents only a small proportion of the theoretical number of proteins encoded by genomic sequences.

To bridge this gap and to meet the demand of rapidly obtaining protein structure information, advancements have been made in SP methodologies in the form of HT technologies. However, these technologies have encountered some of the traditional bottlenecks in structure determination for difficult proteins and complexes of proteins at HT. To overcome these bottlenecks, efforts have been focused on improving the structure determination pipeline by streamlining and optimizing protein production, protein crystallization, data collection and structure solution. In addition, SP centers have adopted bioinformatics analysis of potential targets to generate models based on solved structures and to establish collaborative research to exploit the function of proteins. Recently, in the USA, the National Institutes of Health established a *Protein Structure Initiative*(PSI): a biology network to determine protein structures including membrane proteins of high biological interest. The objective of the PSI is to develop suitable technologies for membrane protein structure solution, using bioinformatics and modeling to leverage solved structures, and to carry out collaborative research to provide a link between a structure and its biomedical and biotechnological impact. On the other hand, in Europe, the emphasis for the *Structural Proteomics IN Europe*(SPINE) initiative has been to apply these HT technologies to systems of biological interest, the ultimate aim being to solve significant biological problems more effectively. Furthermore, the European *INSTRUCT*project offers scientists access to world-class structural biology and SP infrastructures and expertise. *INSTRUCT*makes integration possible more rapidly, creating a coherent forum for structural biology. This forum will stimulate closer collaboration between scientific communities and initiatives in biological sciences.

In this report, recent advances in protein structure analysis in the context of SP will be discussed. Furthermore, the impact of SP on other biological sciences including drug discovery and biotechnology will be explored.

## Automation and strategies for protein structure analysis

### Protein production and crystallization

Generating pure, soluble and homogeneous protein for structure determination is a major rate-limiting step in the overall process. Traditional sequential generation of single expression constructs for a single protein target has been superseded by parallel, HT cloning techniques. Genetic engineering and the use of specific crystallization chaperones are two approaches that have proven invaluable for the determination of many highly important protein structures.

#### Screening of candidate proteins

Structural biology projects are typically initiated to characterize the biological activity of a specific protein or protein complex. In some cases, crystallization of that exact protein leads to a structure that can be correlated directly to functional data. However, in many cases, researchers will eventually come to the conclusion that the protein of interest is not suitable for structural analysis. It makes sense, therefore, to include parallel, HT approaches early on for identifying optimal boundaries and experimental conditions for protein production and crystallization. The selection of candidate proteins is very much project dependent, but will usually include orthologs or homologs of the original protein of interest and genetic constructs corresponding to subregions or individual domains. Methods for HT characterization of larger numbers of expression clones have originally been described for bacterial expression systems (Berrow et al. 2006; Büssow et al. 2005). Small-scale expression testing is more difficult to achieve in eukaryotic systems such as yeast (Holz et al. 2003) and baculovirus (McCall et al. 2005). *Transient transfection*of mammalian cell lines such as HEK293 is a highly efficient system for secreting mammalian glycoproteins and has also been successfully applied to produce membrane proteins such as rhodopsin (Standfuss et al. 2011). This method can be performed in a HT manner for characterization of protein candidates for crystallization (Lee et al. 2009).

#### Glycoproteins

The choice of expression system has a great influence on the quality and quantity of the produced recombinant protein. *Cell-free*protein production has proven its value for producing soluble (Makino et al. 2010) and membrane proteins (Junge et al. 2011; Reckel et al. 2010) for NMR and crystallographic studies (Watanabe et al. 2010). Mammalian proteins stabilized by disulfide bonds and modified by glycosylation are especially demanding targets. Mammalian cells are the ideal host for these proteins, since they yield protein with all the post-translational modifications required for biological activity, including authentic glycosylation and correct disulfide pairing. However, cell culture is time and labor intensive. Many extracellular mammalian proteins can be recovered in active form through refolding of bacterial inclusion body proteins (Vallejo and Rinas 2004). Obviously, these proteins do not require post-translational modifications other than disulfide bridges for correct folding. *Refolding*of proteins from inclusion bodies is common in industrial production but requires extensive process optimization. Structural biology projects applying inclusion body refolding benefit from automated screening of folding conditions with generic, biophysical assays (Cowieson et al. 2006; Scheich et al. 2004; Vincentelli et al. 2004).

**Fig. 3:**
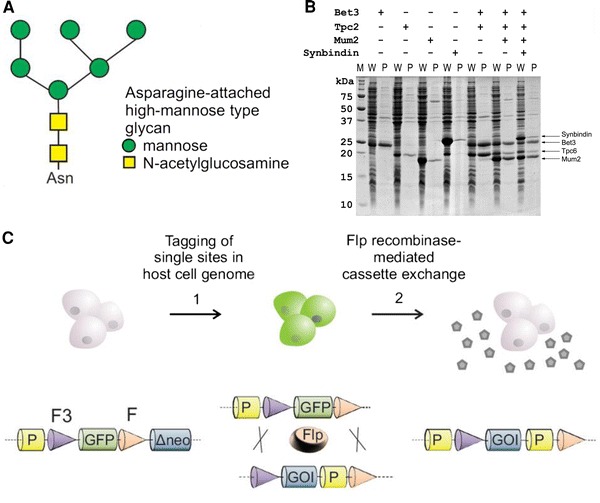
Protein production. **a**Glycosylation: structure of a high mannose-type glycan. **b**Co-expression of a complex of four proteins with the pQLink system.*M*: marker,*W*: whole cellular protein,*P*: purified protein (Scheich et al. 2007). **c**Cell line development by *recombinase-mediated cassette exchange*(RMCE): cells are transfected with a vector containing a*GFP*gene flanked by recombination sites*F3*and*F*and GFP-positive cells are isolated. Cassette exchange is initiated by co-transfecting a tagged cell line with an Flp recombinase expression vector and a targeting vector bearing the gene of interest (GOI). Flp recombinase exchanges the tagging gene cassette and a production cell line is obtained (Wilke et al. 2011)

*Animal cell lines*are highly effective for the secretion of proteins with native glycosylation and disulfide bonds. Glycoproteins produced with the mammalian CHO or HEK293 cell lines carry heterogeneous, complex-type oligosaccharide chains attached to Ser/Thr (O-linked) or Asn (N-linked) side chains. Crystallization of glycoproteins is difficult because of the heterogeneity and flexible conformation of the bulky oligosaccharides, which can also mask possible sites of crystal contacts on the protein surface. Some glycosylation sites can be removed by mutagenesis. Regions with O-linked glycosylation are generally proline-rich and unfolded, and can be excluded from genetic constructs. However, many proteins require glycosylation for folding and transport through the secretory pathway. Enzymatic removal of N-linked glycans from the purified protein with endoglycosidase H or F leaves a single monosaccharide attached, which may increase the solubility of the deglycosylated protein. Enzymatic deglycosylation is efficient for oligosaccharides of the high-mannose type as obtained from the baculovirus system (Fig. 3a). Processing of N-linked glycans by mammalian cell lines results in complex-type oligosaccharides that are difficult to cleave enzymatically. Complex-type glycosylation can be prevented by chemical glycosylation inhibitors (Chang et al. 2007) or by mutating the host cells. The gene for the enzyme *N*-acetylglucosaminyl-transferase I (GnTI), which modifies high-mannose type oligosaccharides, has been mutated in the cell lines CHO Lec1, Lec3.2.8.1 (Stanley 1989) and HEK293S-GnTI^(−)^(Reeves et al. 2002). These cell lines and normal HEK293 cells treated with the glycosylation inhibitors kifunensine or swainsonine have enabled the production of many glycoproteins and their crystallization upon enzymatic deglycosylation (Aricescu et al. 2006; Chang et al. 2007; Davis et al. 1993; Standfuss et al. 2011). Optimized protocols and cell lines allow performing transient transfection of HEK293 at up to liter scale with inexpensive reagents (Aricescu et al. 2006). However, not all proteins can be produced in sufficient amounts by transient transfections. Stable cell lines allow the production of proteins more reproducibly and in much larger volumes in bioreactors. However, establishing lines with good performance requires considerable effort. Novel approaches of *stable cell line*development, based on preparative cell sorting and *recombinase*-*mediated cassette exchange*(*RMCE*Fig. 3c), combine faster development times with improved performance and have been used successfully for X-ray crystallography studies (Wilke et al. 2010, 2011).

#### Crystallization chaperones

Some protein families require a combination of specialized strategies for successful crystallization. Crystallization chaperones are proteins that specifically bind to the target protein and support “carrier-driven” crystal growth (reviewed by (Koide 2009)). They limit the conformational flexibility of the target protein and provide a large, hydrophilic interaction surface for initiating crystal lattice contacts. *Fab fragments*of monoclonal antibodies have been used traditionally as crystallization chaperones. In addition, recombinant antibodies from camels (V_H_Hs, also called “nanobodies”) and synthetic scaffold proteins have demonstrated their usefulness in many examples (Koide 2009). Disulfide-free synthetic scaffold proteins such as *designed ankyrin repeat proteins*(*DARPINs*) or the fibronectin type III domain (FN3) can be screened for specific binders in vitro and can be produced easily in *E. coli*. Fab fragments enabled the first crystal structure to be determined for a non-rhodopsin GPCR (Rasmussen et al. 2007) and a full-length potassium channel (Uysal et al. 2009). Furthermore, the first high-resolution crystal structure of the β2 adrenergic receptor–Gs protein complex has recently been reported in which nanobodies (camelid antibody fragments) were used to significantly improve crystal quality (Rasmussen et al. 2011). Nanobodies are relatively simple proteins, about a tenth the size of antibodies and just a few nanometers in length.

#### Protein engineering

Protein engineering can overcome problems with producing sufficient amounts of protein, keeping protein soluble at the concentrations required for crystallization and obtaining proteins with surfaces that allow crystal formation (Derewenda 2010). In general, protein engineering follows one of three strategies: designing shortened proteins lacking terminal residues outside the globular fold, mutating residues on the target protein’s surface or designing fusion proteins.

If a full-length protein cannot be produced or crystallized, then a common strategy is to design shorter variants which represent isolated domains, eliminating flexible regions at the termini or large internal loops. Databases such as *PFAM*provide information on the presence of *conserved domains*in protein sequences. Alternatively, genetic constructs can be designed that avoid regions predicted to be disordered and unfolded by software tools such as DISOPRED2 (Ward et al. 2004), RONN (Yang et al. 2005), or the meta-server metaPrDOS (Ishida and Kinoshita 2008). The strategy of designing genetic constructs on the basis of computational analysis may fail because of imprecise or missing information in the respective databases. Robotic screening of random truncation libraries represents an alternative technique in such cases (reviewed by (Dyson 2010; Yumerefendi et al. 2011)). In this technique, the cDNA is fragmented and a library of expression clones is created by cloning the fragments. By chance, a few clones of the library will contain a fragment that encodes for just one complete domain. Such clones will express soluble protein. Different ways of screening libraries for clones that express soluble protein have been described, including a filtration technique (Cornvik et al. 2005), the biotinylation assay of the *ESPRIT*technology (Yumerefendi et al. 2011) and screening based on GFP fusion protein fluorescence (Pedelacq et al. 2011). The *ESPRIT*(*Expression of Soluble Proteins by Random Incremental Truncation*) library technology has been adapted recently to allow screening for soluble protein complexes (An et al. 2011).

Single point mutations can have a dramatic effect on a protein’s solubility and crystal formation (Derewenda 2010). The most successful point mutation strategy, *surface entropy reduction*(SER), replaces small clusters of two to three surface residues with high conformational entropy such as Lys, Glu or Gln with Ala. SER produces mutants that are often more susceptible to crystallization than the wild-type protein. More than 100 structures of proteins optimized by SER have been solved. A Web server facilitates protein engineering for SER (http://services.mbi.ucla.edu/SER/). In general, SER does not improve protein solubility. Proteins that cannot be concentrated to sufficient levels for crystal growth benefit from strategies opposite to SER. The solubility of such proteins can often be increased by reducing the hydrophobicity of surface residues. This approach is more difficult than SER, because exchanging hydrophobic surface residues requires some knowledge of the protein’s structure.

Directed point mutations are generally not used to improve the protein production levels since no rational strategies are available. However, screening of large libraries of random mutants of the target proteins, enabled by laboratory automation, has been successful (Cornvik et al. 2005; Listwan et al. 2009; Yumerefendi et al. 2011). Fusion proteins with partners such as glutathione S-transferase (GST), thioredoxin or maltose binding protein (MBP) are a more common approach to improve the target protein’s production and solubility. However, the flexible linker between the fusion partners generally inhibits crystallization. Furthermore, when the fusion partner is removed using a site-specific protease, the improvement in solubility conferred by the fusion partner may be lost. Careful design of MBP fusion proteins enables *carrier*-*driven crystallization*of intact fusion proteins (Moon et al. 2010). These fusions have to be designed in such a way that the MBP’s C-terminal α-helix is fused directly to the globular core of the target protein, thereby avoiding flexibility between the fusion partners. Then, the MBP part can improve protein yield and solubility and promote crystal growth.

One method of surface modification that does not involve additional cloning is reductive lysine methylation, where lysine side chains are chemically modified (Sledz et al. 2010; Walter et al. 2006). The technique can improve the X-ray diffraction of existing crystals, or permit the crystallization of proteins that had previously failed to yield crystals.

#### Protein complexes

Protein complexes are attractive targets for X-ray crystallography, because their structures reveal important information relating to the molecular details of specific protein recognition. However, crystallization of a complex requires careful preparation that includes critical assessment of the available data, careful optimization of sample preparation and functional and biophysical characterization of the complex using a variety of methods (Collinet et al. 2011; Perrakis et al. 2011). Very stable complexes that do not dissociate are preferred targets for crystallization. However, the subunits of such stable heterocomplexes may not be able to fold into a soluble conformation alone, necessitating the *co*-*expression*of the complex components. Transient complexes, on the other hand, which exist in equilibrium with the dissociated subunits, are more difficult to crystallize because of sample heterogeneity. Subunits of transient complexes may form crystals that exclude the other subunit, which is often difficult to detect. Recombinant production of the subunits of a protein complex in the same host cell by co-expression has been described with a large variety of systems (Busso et al. 2011; Nie et al. 2009; Vijayachandran et al. 2011). Novel cloning strategies enable co-expression of many subunits in host cells including *E. coli*, baculovirus and mammalian cells (Berger et al. 2004; Kriz et al. 2010; Trowitzsch et al. 2010), and have been adapted to automated cloning (Bieniossek et al. 2009). The *pQLink*system (Scheich et al. 2007) allows co-expression of an unlimited number of protein subunits in *E. coli*with different affinity tags (Fig. 3b). *pQLink*vectors have been widely used by different laboratories, mainly for eukaryotic vesicle tethering complexes (Kummel et al. 2008; Lees et al. 2010; Ren et al. 2009). Studies comparing a large variety of expression systems have demonstrated that subtle changes in the expression strategy have a profound effect on the success of co-expression experiments, even if the main parameters, protein sequence and host cell are identical (Busso et al. 2011).

Successful recombinant expression of protein complexes requires that the subunits are synthesised in similar amounts. Otherwise, the yield of the complete complex is determined by the subunit present in the lowest concentration. Also, a heterogeneous mixture of the complete complex with smaller oligomers not comprising all subunits is obtained. To circumvent this problem, the synthesis of *polyproteins*has been introduced for generating protein complexes (Vijayachandran et al. 2011). This strategy is reminiscent of the genomes of many viruses that contain large open reading frames encoding *polyproteins*that are cleaved by viral proteases into single proteins upon translation. A baculovirus vector containing a large open reading frame comprising single protein sequences separated by a site-specific protease site was created. The coding sequence of the TEV protease was included in the vector. Upon overexpression, intracellular TEV protease cleaved the polyprotein into single subunits of a protein complex. This strategy was successfully demonstrated for sub-complexes of human general transcription factor TFIID and other complexes (Vijayachandran et al. 2011).

#### Protein crystallization methods and automation

Production of protein crystals suitable for structural studies poses one of the major bottlenecks in the entire process. Finding crystallization conditions that yield single, well-ordered crystals with low mosaicity that diffract to sufficient resolution can be very challenging. The quality of a crystal is often linked to the number of crystals formed (a few large crystals versus many microcrystals), size (larger is better) and appearance (optically clear, sharply faceted crystals are best). However, any true measure of quality must verify that the diffraction properties correlate with the morphological quality of the crystal.

Crystallization can occur spontaneously, or alternatively it can take several days, weeks or months for crystals to appear. Longer crystallization times are usually indicative of proteolytic cleavage at the protein termini promoting crystal formation. It is not easy to provide an estimate for maximum protein crystal growing time. There have been some reports showing that, in some cases, protein crystals may take as long as 6 months or a year to appear. However, an average growing time for a protein crystal is typically less than a month. Normally, protein crystallization occurs when the concentration of protein in solution is greater than its limit of solubility, so that the protein solution becomes supersaturated. To crystallize a protein, it undergoes slow precipitation from an aqueous solution. As a result, individual protein molecules align themselves in a repeating series of “unit cells” by adopting a uniform orientation. One unavoidable aspect of crystallizing a newly expressed protein is the need to carry out a large number of experiments to find suitable conditions in which the protein crystallizes. It can be extremely tedious and time consuming to set up a broad array of different crystallization experiments manually. With the advent of HT liquid handling and crystallization systems, it is relatively easy to prepare a thousand or more crystallization experiments in which crystallization parameters, such as the ionic strength, pH, protein and precipitant concentration and temperature, are varied systematically. However, the success rate does not depend upon the number of crystallization conditions tested.

**Fig. 4:**
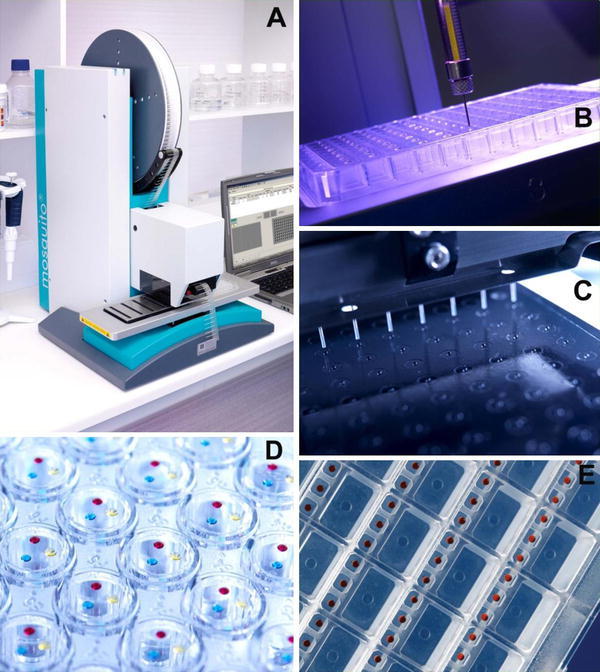
Protein crystallization and automation. **a**TTP LabTech’s mosquito^®^Crystal automates protein crystallography vapor diffusion set-ups, additive screening and microseeding; **b**TTP LabTech’s mosquito^®^LCP: a dedicated instrument for crystallising membrane proteins using lipidic cubic phase screening. The panel highlights the positive displacement syringe, which dispenses the highly viscous lipid mesophases used in the LCP technique into 96-well crystallization plates. (**c**and **d**) Crystallization plate set up for hanging drop vapor diffusion experiments; **e**nanoliter sitting drop experiments set up in a 96-well plate. (*Images courtesy of TTP LabTech Ltd, UK*)

Methods used for crystallization include vapor diffusion, batch crystallization, dialysis, seeding, free-interface diffusion and temperature-induced crystallization. The most popular method for setting up crystallization experiments is vapor diffusion, which includes hanging drop (for smaller volumes), sitting drop (for larger volumes), the sandwich drop, reverse vapor diffusion and pH gradient vapor diffusion methods. A drop containing a mixture of precipitant and protein solution is sealed in a chamber with pure precipitant. Water vapor subsequently diffuses from the drop until the osmolarity of the drop and the precipitant is equal. The dehydration of the drop causes a slow concentration change of both protein and precipitant until equilibrium is achieved, ideally in the crystal nucleation zone of the phase diagram (Dessau and Modis 2011). Batch crystallization relies on bringing the protein directly into the nucleation zone by mixing protein with the appropriate amount of precipitant. The batch method is usually carried out under oil to prevent the diffusion of water out of the drop (Chayen 1997). Many of these methods can be performed using HT automated instrumentation and miniaturization of crystallization experiments and have had huge impacts on protein crystallization in terms of saving time and conserving precious sample. For example, crystallization robots such as the *Phoenix*™ *RE*(Rigaku Corporation) and the *Mosquito*^*®*^(TTP Labtech), which can accurately and reproducibly dispense very small volumes (nl in size) into 96-well plates for automated screening and optimization of crystallization conditions, are now commonplace in many laboratories (Fig. 4). In addition, TTP LabTech’s *Mosquito*^*®*^*LCP*(Lipid Cubic Phase) has been designed to aid in the crystallization of membrane proteins by accurately dispensing nanoliter quantities of highly viscous lipids or detergents that are required to retain the structural integrity of the sample. A recent development in protein crystallization has been the use of high-density, chip-based microfluidic systems for crystallizing proteins using the free-interface diffusion method at nanoliter scale, including Emerald Biosystems *MPCS*(Microcapillary Protein Crystallization System)(Gerdts et al. 2008), Fluidigm Corporations *TOPAZ*^*®*^system (Segelke 2005) and the Microlytic *Crystal Former*(Stojanoff et al. 2011). These platforms have the advantage of using minimal protein sample to screen a broad range of crystallization conditions. The Rigaku CrystalMation™ system was set up to fully automate the crystallization process while dealing with sample volumes of 100 nl per experiment.

A popular strategy for the optimization of crystallization conditions in vapor diffusion is crystal seeding. Seeding decouples nucleation from crystal growth and involves transferring previously obtained seed crystals into undersaturated drops. Homogeneous seeding techniques include microseeding, streak seeding and macroseeding. Seed stock for microseeding can be conveniently generated using Hampton Research’s Seed Bead kit. More recently, a simple, automated microseeding technique based on microseed matrix screening has been developed (D’Arcy et al. 2007). This method consists of the addition of seeds into the coarse screening procedure using a standard crystallization robot and has been shown to not only produce extra hits, but also generate better diffracting crystals. Successful cases for a simple semi-automated microseeding procedure for nanoliter crystallization experiments have also been recently described (Walter et al. 2008). Furthermore, crystallization plate storage and inspection are now fully automated. For example, the *Minstrel™ drop imager family*(Rigaku) and *Rock Imager*(Formulatrix) combine imagers with gallery plate hotels/incubators to store crystallization plates at a constant temperature, periodically inspect them and manage the data (Hiraki et al. 2006; Walter et al. 2005).

Despite the progress that has been made in increasing throughput, the act of identifying crystals in the crystallization experiments remains a task requiring human intervention. A number of attempts are being made to automate crystal detection from the imaged drop and varying degrees of success have been reported (Liu et al. 2008). Automated crystal recognition has the potential to reduce the time-consuming human effort for screening crystallization drop images. Several approaches have been suggested to increase contrast for imaging and detection of protein crystals in such cases: crystal birefringence (Echalier et al. 2004), addition of fluorescent dyes (Groves et al. 2007) and monitoring the fluorescence of trace labeled protein molecules (Forsythe et al. 2006). The identification of crystallization hits has been simplified by UV detection combined with conventional imaging (Judge et al. 2005). For example, the latest generation of imaging systems combine visible and UV inspections providing a powerful tool for monitoring crystallization trials: when crystals are still too small to be mounted, the intrinsic protein fluorescence signal gives confidence that a crystallization hit is worth pursuing. *Second*-*order nonlinear optical imaging of chiral crystals*(*SONICC*) is an emerging technique for crystal imaging and characterization (Kissick et al. 2010, 2011). SONICC imaging has been found to compare favorably with conventional optical imaging approaches for protein crystal detection, particularly in non-homogeneous environments that generally interfere with reliable crystal detection by conventional means.

A recent development is the *X*-*CHIP*(X-ray Crystallization High-throughput Integrated Platform): a novel microchip that provides a stable microbatch crystallization environment and combines multiple steps of the crystallographic pipeline from crystallization to diffraction data collection onto a single device (Kisselman et al. 2011). This system facilitates HT crystallization screening, visual crystal inspection, X-ray screening and data collection. The chip eliminates the need for manual crystal handling and cryoprotection of crystal samples while allowing data collection from multiple crystals in the same drop.

### Data collection and processing

Data acquisition involves the recording of a series of X-ray diffraction images using a detector. The process of crystal mounting, centering, exposing with X-rays, recording diffraction data and dismounting the crystal represent the major steps in crystallographic data collection.

#### Radiation source, crystal handling and detector: past and present tools

**Fig. 5:**
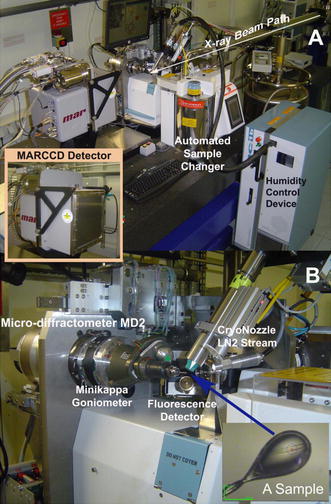
X-ray data collection facility. **a**End-station instrumentation at ESRF beamline BM14 (http://www.bm14.eu) illustrating the sample changer used to exchange cryo-frozen crystals on the goniometer and the MARCCD (Marresearch GmbH) detector used to collect diffraction images. The*arrow*highlights the path of the X-ray beam. **b**Close-up view showing the frozen crystal sample in the center of the image and the surrounding beamline instrumentation. The *red cross*and *blue circle*represent the center and diameter of the X-ray beam on the frozen crystal sample (*bottom right*)

In the past, protein crystals typically ranging in size from tenths of a millimeter to several millimeters were mounted in glass capillary tubes. To collect data, the capillary tube was mounted on a goniometer and exposed to X-rays at room temperature. These X-rays were generated by low flux, sealed tube sources. Nowadays, data collection is handled by automated sample changers and micro-diffractometers in a cryogenic (100 K) environment utilizing brighter synchrotron radiation as the X-ray source. Cryo-freezing the sample inhibits free radicals diffusing through the crystal during data collection: these free radicals cause secondary radiation damage that leads to degradation in the quality of collected data. There are currently in excess of 125 dedicated protein crystallography beamlines around the World. The X-ray films that were used for data recording in the past have now been superseded with charge-coupled devices (CCD) and pixel array detectors, which allow diffraction data to be recorded directly and stored straight to disk. For example, a recent development has been the *PILATUS*detector (*pixel apparatus for the SLS*), which has no readout noise, superior signal-to-noise ratio, a readout time of 5 ms and high dynamic range compared to CCD and imaging plate detectors. Delivery of high flux beam at third-generation synchrotron sources coupled with the advances in detector technology and control systems have significantly accelerated the speed of macromolecular diffraction data collection. An example of a state-of-the-art synchrotron X-ray data collection setup is shown in Fig. 5. Nowadays, crystals larger than 50 μm in size can be evaluated at conventional synchrotron beamlines. However, with some targets such as membrane proteins and multi-protein complexes, it is notoriously difficult to obtain crystals of sufficient size and order to generate high-quality diffraction data. Hence, next generation microfocus beamlines with reduced beam sizes have been established at synchrotron sites around the world, allowing measurements to be made on crystals a few micrometers in size. It has been predicted that a complete data set with a signal-to-noise ratio of 2σ at 2 Å resolution could be collectable from a perfect lysozyme crystal measuring just 1.2 μm in diameter using a microfocus beam (Holton and Frankel 2010). A number of crystal structures have been solved using micrometer-sized crystals by merging data from several crystals, including a polyhedron-like protein structure (~5–12 μm) (Coulibaly et al. 2007) and a thermally stabilized recombinant rhodopsin (with crystal dimensions of 5 × 5 × 90 μm^3^) (Standfuss et al. 2007). Recently, strategies have been developed to determine structures from showers of microcrystals using acoustic droplet ejection (ADE) to transfer 2.5 nl droplets from the surface of microcrystal slurries through the air and onto micromesh loops. Individual microcrystals are located by raster-scanning a several-micron X-ray beam across the cryocooled micromeshes. X-ray diffraction data sets are subsequently merged from several micrometer-sized crystals and this technique has been used to solve 1.8 Ǻ resolution crystal structures (Soares et al. 2011).

As a result of these technological advancements, the time required to setup a diffraction experiment has become a significant proportion of the total time of an experiment. The diffraction experiment involves sample mounting, crystal centering and determination of data collection parameters. Significant progress has been made in automating crystal mounting, crystal centering and the energy scan to find metals or ions present in crystals that can be used for phasing (Heinemann et al. 2003; Shi et al. 2005). Automated sample mounting systems allow users to mount samples on the beamline without entering the experimental hutch. These systems minimize the need for manual intervention and facilitate the rapid and systematic screening of dozens of samples. For example, the automated sample changers equipped at the EMBL/ESRF beamlines are capable of handling 50 frozen samples, whereas the *ACTOR*(*Automated Crystal Transfer, Orientation and Retrieval*) robots installed on the beamlines at the DIAMOND synchrotron can mount up to 80 cryogenically frozen samples from their onboard storage dewars. This facilitates the rapid screening and ranking of crystals and enables users to collect data from their best diffracting crystal(s) (Beteva et al. 2006; Cipriani et al. 2006). These features make the automated approach far quicker than manual operations insuring that beamtime is used efficiently.

Before starting data collection, the crystal needs to be aligned so that it is coincident with the X-ray beam and the rotation axis. This is normally performed manually by the user at the beamline. However, for fully automated operation of the beamline, automated crystal centering is a prerequisite, especially when sample mounting robots are used. Semi-automated crystal centering based on a user clicking a mouse to indicate the position of the crystal through a specially designed software interface has been shown to be relatively robust and is employed at most synchrotron beamlines (Snell et al. 2004). Recent reports show that it is possible to center crystals automatically without user intervention using the recognition software *C3D*(Lavault et al. 2006), *XREC*(Pothineni et al. 2006) or alternatively by using the diffraction method (Hilgart et al. 2011; Song et al. 2007). Crystal centering based on the diffracton method is especially attractive for micrometer-sized crystals. Optical centering of small crystals is challenging since visible light wavelengths (0.4–0.7 μm) are comparable to the crystal size and many crystals have irregular diffraction quality, which cannot be addressed by this technique. In diffraction-based crystal centering, the crystal is scanned in two dimensions using a small step size and at each step a diffraction image is taken, which is analyzed for locating and counting diffraction spots. The scored results are presented in a table which allows users to select optimally diffracting areas within the macroscopic sample (Cherezov et al. 2009).

#### Data collection and processing software packages

**Fig. 6:**
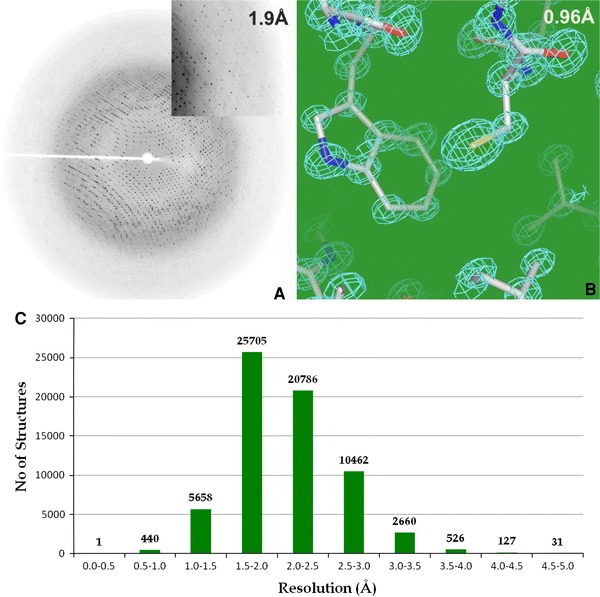
Accuracy and details. **a**Representative X-ray diffraction pattern collected on a Marresearch GmbH imaging plate system. The diffraction extends to a maximum of 1.9 Å resolution at the edge of the image. **b**Representative portion of an electron density map at 0.96 Å resolution. The sticks represent the individual atoms for the amino acids that constitute the protein (carbon, *gray*; nitrogen, *blue*; oxygen, *red*; sulfur, *yellow*) and the chicken wire represents the corresponding experimental electron density for these atoms. **c**Histogram depicting the distribution of resolutions for protein structures in the PDB as of August 2011

Typically, data extending to 2.5 Å resolution or higher are desirable for novel proteins and protein–ligand complexes, so that the model can be fitted unambiguously into the electron density map. However, in more challenging cases, data at 3 Å resolution or lower may be sufficient to fit the overall fold of a protein or the constituents of a multi-protein complex. A typical X-ray diffraction image, the electron density map to atomic resolution and the distribution of resolutions for protein structures in the PDB are depicted in Fig. 6. However, in many cases, diffraction properties of crystals are not known in advance, especially when crystals are small (in the micrometer range) and cannot be prescreened using in-house instrumentation prior to a synchrotron trip. It often takes a significant amount of time at the synchrotron to screen these sub-micron crystals to identify a well-diffracting crystal suitable for data collection. Whilst collecting data at the synchrotron beamline, the user must make decisions about the parameters of the experiment—exposure time, rotation range, oscillation angle, detector distance, beam size and wavelength—based on their experience, visual inspection of the diffraction images and information output by data-processing packages. Most of the instrumentation in the experimental station is computationally controlled using software packages such as *Blu*-*Ice*(McPhillips et al. 2002), *CBASS*(Skinner et al. 2006), *MxCube*(Gabadinho et al. 2010) and *JBlue*-*Ice*(Stepanov et al. 2011). However, very often an intuitive decision is made by the user on the exposure time to use. In cases where this has been overestimated, it can lead to significant radiation damage before the completion of data collection. In addition, an inappropriate data collection strategy can lead to the failure of an experiment. Computationally efficient modeling of the data statistics for any combination of data collection parameters provides a foundation for making a rational choice. The modeling of data statistics using a few test images allows one to quantitatively select which screened crystal gives the highest resolution using an appropriate rotation range and X-ray radiation dose prior to data collection (Bourenkov and Popov 2006, 2010).

The evaluation of the collected reflection intensities on the diffraction images involves the integration of the total intensity within all pixels of the individual spot profiles. The crystallographic program *HKL2000*is capable of carrying out data processing automatically (Borek et al. 2003; Minor et al. 2006). Other commonly used data-processing packages include *XDS*(Kabsch 1993) and *MOSFLM*(Leslie 2006). These programs all give excellent results with high-quality diffraction data, although their treatment of imperfect data differs owing to different approaches to indexing, spot integration and the treatment of errors. These programs can process data from a wide variety of modern area detectors from manufacturers including MarResearch, Rigaku/MSC, ADSC and MacScience. All these programs require crystallographers to make informative decisions and to input the correct experimental parameters to process the data successfully. There are ongoing activities at several synchrotron beamlines to develop expert systems that aim to automate the data collection strategy using the software *BEST*(Bourenkov and Popov 2006), *RADDOSE*(Paithankar and Garman 2010), *MOSFLM*and *XDS*to reduce the time required to successfully collect high-quality X-ray data.

#### Post-crystallization treatments to improve the quality of diffraction

Among the biggest problems in macromolecular crystallography is the relatively weak diffraction power of protein crystals and their sensitivity to ionizing radiation damage. Cryogenic methods provide great advantages in macromolecular crystallography, especially when synchrotron radiation is used for diffraction data collection. Apart from reducing the problem with radiation damage and enabling the storage and safe transport of frozen crystals, there are a number of additional benefits. For example, cryo-freezing can be exploited to trap normally unstable intermediates in enzyme-catalyzed reactions to permit their characterization. In addition, cryo-freezing can dramatically improve diffraction properties by reducing thermal vibrations and conformational disorder within the crystal, provided that the crystal is amenable to freezing and a suitable cryoprotectant has been selected. Of primary practical importance is the decrease in secondary radiation damage in the crystal caused by the diffusion of free radicals, typically permitting a complete data set to be collected from a single crystal. Cryogenic data collection has allowed efficient phasing using multi-wavelength methods.

When a crystal of a biological macromolecule is cooled to cryogenic temperatures, the main difficulty is to avoid the crystallization of any water present in the system, whether internal or external. Therefore, a cooling procedure has to be chosen that leads to a glass-like amorphous phase of the solvent. In principle, there are four options: (1) cooling on a timescale too fast for ice formation to occur (Hartmann et al. 1982), (2) cooling at high pressure by which the formation of the common hexagonal form of ice is circumvented (Thomanek et al. 1973), (3) replacing the liquid surrounding the crystal with a water-immiscible hydrocarbon oil such as *paratone*-*N*(Hope 1988, 1990), *paraffinoil*(Riboldi-Tunnicliffe and Hilgenfeld 1999) and *LV CryoOil*™ (MiTeGen), (4) modifying the physicochemical properties of the solvent by the addition of cryoprotectants in a way that a vitrified state can be reached at moderate cooling rates.

To prevent the nucleation of ice crystals, the last method is currently the most widely used. The crystal is permeated with a diffusible solvent containing cryoprotectants such as glycerol, sucrose or other organic solvents (Garman 1999; Garman and Owen 2005; Heras and Martin 2005). Determining the initial and optimal cryoprotectant concentration is often a process of trial and error. One must find suitable cryoprotectant concentrations that do not destroy the crystalline order while, at the same time, allowing the solvent to form an amorphous glass upon rapid cooling. Recently, trimethylamine *N*-oxide (TMAO) has been shown as a very versatile cryoprotectant for macromolecular crystals (Mueller-Dieckmann et al. 2011).

It has been shown that diffraction properties of flash-cooled macromolecular crystals can often be improved by warming and then cooling a second time—a procedure known as crystal annealing. Two different crystal-annealing protocols have been reported (Garman 1999; Harp et al. 1998, 1999; Samygina et al. 2000; Yeh and Hol 1998) and many variants of these have been tried in the field. The first method involves removing a flash-cooled crystal from the cold gas stream and placing it in a cryoprotectant solution (either glycerol, MPD or Paratone-N oil) for several minutes before refreezing (Harp et al. 1998, 1999). There are several examples cited in the literature where this technique has been successfully applied (Felts et al. 2006; Manjasetty et al. 2001). In the second method, the cold stream is blocked for a fixed amount of time before the crystal is allowed to re-cool (Yeh and Hol 1998). Both annealing protocols can improve crystal resolution and mosaicity, although substantial crystal-to-crystal and molecule-to-molecule variability has also been observed. Recently, the flash annealing technique has been automated using a cryo-shutter (Vahedi-Faridi et al. 2005), a device that blocks the 100 K nitrogen stream that bathes the crystal for a specific amount of time. The main advantage of the shutter system is that it allows a controlled, instant re-cooling of the crystal and the user can perform the flash annealing experiment remotely without entering the experimental hutch.

Diffraction quality can also be improved by post-crystallization treatments, such as controlled dehydration (Heras et al. 2003), to attempt to improve the crystal diffraction properties. A user-friendly apparatus for crystal dehydration has been designed and implemented at the ESRF/EMBL beamlines (Russi et al. 2011; Sanchez-Weatherby et al. 2009). In addition, Proteros biostructures GmbH has developed a *Free Mounting System*(*FMS*™) that precisely controls the humidity around a crystal, which can lead to dramatically improved diffraction data.

#### Remote data collection

Synchrotron data collection can be performed remotely from home institutions by accessing the instrumentation via advanced software tools that enable the network-based control of beamlines (Gonzalez et al. 2005). Remote access to synchrotron sources is becoming more popular, since it saves both time and resource and results in more efficient use of the beamtime. “Mail-in” crystallography (diffraction data measured by synchrotron staff) is another popular option for X-ray diffraction data collection, whereby users ship their crystals to the synchrotron for data collection by the beamline scientists.

### X-ray structure determination

Amplitudes or intensities can be measured directly from the X-ray diffraction experiment, but information relating to their relative phases cannot be measured. To be able to calculate an electron density map and subsequently determine the protein structure, an estimate of the phases has to be obtained indirectly using mathematical approaches and this represents the *phase problem*in protein crystallography.

#### Structure determination methods

**Fig. 7:**
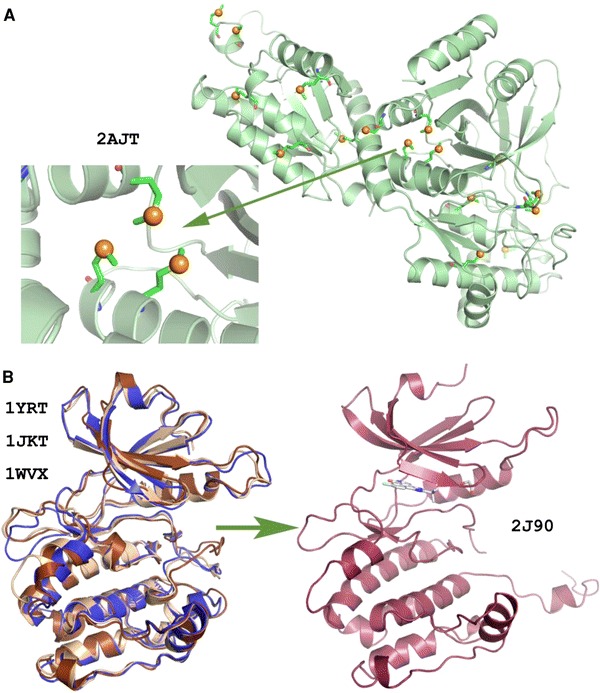
Examples for widely used structure determination methods. **a**Structure of *E. coli*Arabinose Isomerase (PDB 2AJT) determined by *single*-*wavelength anomalous diffraction*(SAD). Selenomithionine residues are also shown (Manjasetty and Chance 2006). **b**Structure of DAPK3 (PDB 2J90) determined with the molecular replacement (MR) method using the template prepared by homolog structures (PDB 1YRT, 1JKT, 1WVX) (Pike et al. 2008)

Heavy-atom incorporation (isomorphous replacement, anomalous scattering and anomalous dispersion), molecular replacement and direct methods are commonly used techniques to solve protein structures. The general requirement for the exploitation of the anomalous signal for the determination of phase estimations via *multiple*or *single*-*wavelength anomalous diffraction*(*MAD*or *SAD*) techniques is that the protein crystal should contain anomalously scattering atoms, e.g., Hg, Pt or Se. With the advent of tunable X-ray sources and improved data collection techniques, it is now possible to measure the intensities of diffracted X-rays with very high precision. The small differences in intensities between Bijovoet pairs due to the presence of heavy atoms can be used to calculate initial estimates of the protein phase angle. One of the strategies widely used for the determination of novel protein structures is selenomethionine incorporation, where selenomethionine is replaced by methionine in the protein during expression. This method has revolutionized protein X-ray crystallography and it is estimated that over two-thirds of all novel crystal structures have been determined using either Se-SAD or Se-MAD (Fig. 7a). Novel structures can also be solved using the weak anomalous signals from atoms, such as sulfur and phosphorous present in certain macromolecules. SAD represents the most commonly used technique for novel proteins in SP centers. *Multiple*or *single isomorphous replacement*(*MIR*or *SIR*) methods also require the introduction of heavy atoms such as mercury, platinum, uranium or gold into the macromolecule under investigation. These heavy atoms must be incorporated into protein crystals without disrupting the lattice interactions so that it remains *isomorphous*with respect to the native crystal. In the *SIR*method, intensity differences between the heavy-atom derivatized and native crystal are used to calculate experimental phases. Recently, the *SIR*phasing protocol has been re-applied in the *radiation damage*-*induced phasing*(*RIP*) technique, where the differences in intensities induced by radiation damage are used as a phasing tool (Ravelli et al. 2003). Limitations of these phasing protocols are mainly due to the deleterious effect that a high X-ray dose has on a protein crystal. X-ray radiation damage induces many changes to the protein structure and to the solvent, resulting in a consistent number of damaged sites and a decrease in the diffraction quality of the crystal. As an alternative to X-rays, *ultraviolet*(*UV*) radiation has been used to induce specific changes in the macromolecule, which only marginally affects the quality of the diffraction (Nanao and Ravelli 2006) while inducing more selective changes to the protein structure. This method is known as *UV*-*RIP*(*ultraviolet radiationdamage*-*induced phasing*). The most striking effect of UV radiation damage on protein crystals, as for X-ray radiation, is the breakage of disulfide bonds. Furthermore, this technique has been extended to a non-disulfide-containing protein, photoactive yellow protein, which contains a chromophore covalently attached through a thioester linkage to a cysteine residue (Nanao and Ravelli 2006) and to selenomethionine (MSe) proteins (Panjikar et al. 2011). Therefore, this method offers considerable potential, and selenium-specific UV damage could serve as an additional or even an alternative way of experimental phasing in macromolecular crystallography (de Sanctis et al. 2011). Another popular method adopted at SP centers is the use of iodide ion soaks and *SAD*experiments for de novo phasing (Abendroth et al. 2011).

*Molecular replacement*(*MR*) requires a search model for the protein under investigation, either determined from X-ray crystallography or from homology modeling, to calculate initial estimates of the phases of the new structure. The use of MR has become more commonplace with the expansion of the PDB and is currently used to solve up to 70% of deposited macromolecular structures where a homolog structure already exists (Fig. 7b: Pike et al. 2008). In cases when there are up to four molecules in the asymmetric unit of the crystal, the search model is structurally similar to the target protein and its oligomeric state is known, the MR method is fairly straightforward using programs such as *MOLREP*(Vagin and Teplyakov 1997), *AMoRe*(Navaza 2001) and *Phaser*(McCoy et al. 2007). To further streamline the MR procedure, a number of automated MR pipelines have been developed. These include the Bias Removal Server (Reddy et al. 2003), *CaspR*(Claude et al. 2004), *BRUTEPTF*(Strokopytov et al. 2005) and MR pipeline (Schwarzenbacher et al. 2008). Other developments include *Auto*-*Rickshaw*(Panjikar et al. 2005) which is principally used for experimental phasing, but also uses phased MR as well as enabling a standard MR phasing protocol using *BALBES*(Long et al. 2008), *MrBUMP*(Keegan and Winn 2008) and a scheme for using comparative models in MR (Raimondo et al. 2007). Recently, MR phasing has been demonstrated for 2.0 Å data based on the combination of localizing model fragments such as small helices with *Phaser*and density modification with *SHELXE*(Rodriguez et al. 2009). In addition, improved MR by density-and energy-guided structure optimization has also been described (DiMaio et al. 2011).

It is worth noting that if an MR search is difficult primarily because the model is extremely poor and the resolution of the X-ray data is limited (lower than 2.0 Å), then the time spent attempting to obtain a solution with that model is usually inversely proportional to the usefulness of the solution once it has been obtained. This is partly because the model suffers from bias and often requires iterative, time-consuming manual correction using computer graphics in combination with model refinement. Interestingly, the determination of the substructure becomes easier when an anomalous difference Fourier synthesis can be calculated using preliminary phases from an MR solution. The subsequent use of this substructure to generate an unbiased electron density map (Baker et al. 1993) is often referred to as *MRSAD*(*molecular replacement with single-wavelength anomalous dispersion*) (Schuermann and Tanner 2003). A combination of MR and SAD has been automated and incorporated into the structure determination platform *Auto*-*Rickshaw*. The complete *MRSAD*procedure includes MR, model refinement, experimental phasing, phase improvement and automated model building; it has been shown that poor MR or SAD phases with phase errors larger than 70° can be improved using this described procedure (Panjikar et al. 2009) and a large fraction of the model can be determined in a purely automatic manner from X-ray data extending to better than 2.6 Å resolution.

#### Computational resources for structure determination

New software packages have been developed for determining the 3D structure of proteins to meet the HT requirements of SP projects (Jain and Lamour 2010). The software pipelines have varying degrees of automation deriving from different aims, but all require minimum user input to facilitate the automated location of heavy-atom sites, phase determination and phase improvement by solvent flipping/flattening, model building and refinement. Ideally, the structure solution process should be carried out in parallel with data processing. For instance, the most recent iteration of the *HKL*suite, *HKL*-*3000*, is capable of integrating automated data collection, processing, structure solution and refinement steps (Minor et al. 2006). The *Auto*-*Rickshaw*suite incorporates many widely used programs for automatic protein structure determination (Panjikar et al. 2005). *AutoSHARP*(Vonrhein et al. 2007), *CRANK*(Ness et al. 2004; Pannu et al. 2011), *BnP*(Weeks et al. 2002), *HKL2MAP*for *SHELX*(Pape and Schneider 2004; Schneider and Sheldrick 2002) and the *PHENIX*suite (Adams et al. 2004) are highly automated and provide all the tools necessary to proceed from substructure solution and phasing through to displaying and interpreting the resultant electron density map. In addition, these programs provide automated protocols to enable protein models to be built rapidly without user intervention, providing feedback on the success of the experiment while the crystal is still at or near the beamline. *AutoSHARP*includes various *CCP4*(Collaborative Computational Project, number 4) programs (Winn et al. 2011), uses the *SHELXD*software for locating heavy atoms and carries out density modification using either *DM*(Cowtan and Zhang 1999) or *SOLOMON*(Abrahams and Leslie 1996), while *ARP/wARP*(Langer et al. 2008; Morris et al. 2003) or *BUCCANEER*(Cowtan 2006) are used for automated model building. This pipeline can be run without user intervention once suitable input has been provided and can be rerun from any of the structure solution steps by the user whenever desired. The *CRANK*package invokes *BP3*(Pannu and Read 2004), *CRUNCH2*(de Graaff et al. 2001), *SHELXD*, *SOLOMON*, *DM*, *RESOLVE*(Terwilliger 2000), *BUCANEER*and *ARP/wARP*along with a few *CCP4*programs and uses standard XML input at every step of the structure solution. The process may be invoked either using the *CCP4*graphical user interface (*CCP4i*) or off-line and the user must choose the defined path through the pipeline. The *BnP*pipeline includes *SnB*(Xu and Weeks 2008) and the *PHASE*package for structure solution. *HKL*-*3000*is a commercially available software package, which includes the data-processing programs *DENZO/SCALEPACK*(Otwinowski and Minor 1997) along with structure solution programs including modified versions of *MLPHARE*, *SHELXC/D/E*, *DM*and *ARP/wARP*. The *PHENIX*software suite is a highly automated system for macromolecular structure determination that can rapidly arrive at an initial partial model of a structure without significant human intervention, given moderate resolution and good quality data. The *Auto*-*Rickshaw*pipeline has been developed with its primary aim to validate the X-ray diffraction experiment while the crystal is still at or near the synchrotron beamline. The software pipeline is optimized for speed so that the user has the ability to evaluate their data in the minimum possible time. *Auto*-*Rickshaw*makes use of publicly available macromolecular crystallography software. The entire process in the pipeline is fully automatic. Each step of the structure solution process is governed by the decision-making module within *Auto*-*Rickshaw*, which attempts to mimic the decisions of an experienced crystallographer for a number of phasing protocols (SAD, MAD, RIP, MR and variations thereof). Once the input parameters (number of amino acids, heavy atoms, molecules per asymmetric unit, probable space group and phasing protocol) and X-ray intensity data have been input into *Auto*-*Rickshaw*, no further user intervention is required. It proceeds step by step through the structure solution using the decision makers. In cases where a problem is encountered during the structure solution process, the user is informed so that the data collection, the data quality, space group ambiguity or optimization of the anomalous signal is flagged as a problem. Once all the steps have been run successfully, *Auto*-*Rickshaw*provides a tarball, which includes all the necessary files to evaluate the electron density map and model, including ready-made scripts for the graphics programs *COOT*(Emsley and Cowtan 2004; Emsley et al. 2010), *O*(Jones et al. 1991) and *XtalView*(McRee and Israel 2008). The *Auto*-*Rickshaw*server (http://www.embl-hamburg.de/Auto-Rickshaw) is freely accessible to the SP community to aid in their protein structure determination effort.

### Structure and function

Proteins play key roles in almost every biological process and participate in a variety of physiological functions. The key to unraveling how proteins perform their different roles lies in understanding the relationship between protein structure and function.

#### Computational tools and Web servers

A wiki, *Proteopedia*, provides the forum to contribute and share information about a particular 3D structure to exploit its functional role through collaboration, follow-up studies and joint publication (Hodis et al. 2010; Prilusky et al. 2011). Many of the protein structures determined at SP centers are ‘hypothetical proteins’ of unknown function. Insights into the biochemical function of these proteins can be derived by bioinformatics tools such as *TOPSAN*(*The Open Protein Structure Annotation Network*), a Web-based platform which facilitates collaborations that have resulted in insightful structure–function analysis for many proteins leading to numerous peer-reviewed publications (Ellrott et al. 2011; Weekes et al. 2010). A number of other servers, such as *ProFunc*(Laskowski et al. 2005), *3D*-*Fun*(von Grotthuss et al. 2008) and *ProTarget*(Sasson and Linial 2005), enable automated protein function annotations using protein structures. These servers accept protein coordinates in the standard PDB format and compare them with all known protein structures in the PDB. If structural hits are found for proteins of known function, they are listed together with their function and some vital comparison statistics. A recently developed platform called iSee (*interactive Structurally enhanced experience*) allows the interaction and annotation of novel structures and provides a powerful tool for disseminating the full range of structural information. Interestingly, this platform is hosting and featuring the animations adopted by journals that ‘fly’ the reader through structural representations to specific molecular features (Davis et al. 2010; Rellos et al. 2010). This novelty in exposing structural knowledge across the life sciences is one of the breakthroughs attributable to SP.

#### Protein fold, sequence motif, binding site, oligomeric state and surface features for protein annotation

The 3D folding patterns of proteins can be categorized into classes which allow inferences to be made about their molecular function. A comparison of the 3D structures of proteins of known function in the PDB has shown that proteins sharing common folds are often evolutionarily related. *Protein folds*are more highly conserved over time than protein sequence, and structural similarities between a protein of known function and a novel (hypothetical) protein implies that these proteins have related function (Chothia and Lesk 1986). The most commonly used servers for comparing the fold of a newly determined structure against structures in the PDB are *DALI*at EMBL (Holm and Sander 1999) and *VAST*(*Vector Alignment Search Tool*) at NCBI (Gibrat et al. 1996). For example, the hypothetical protein FLJ36880 has a fold which indicates that it is a member of the fumarylacetoacetate hydrolase family (Manjasetty et al. 2004b) and the fold of MJ0882 highly resembles that of a methyltransferase despite limited sequence similarity to any known methyltransferase (Huang et al. 2002). *Structural motifs*in a protein usually represent regions characteristic of specific functions (Godsey et al. 2007). For example, helix–turn–helix motifs are highly conserved in DNA binding proteins. Similar functional sites can be found across different folds in proteins as a result of convergent evolution. This is frequently observed across folds involving *metal ions*. The server *PINTS*(*Patterns in Non*-*homologous Tertiary Structures*) helps to identify functional patterns in non-homologous structures (Stark and Russell 2003). For example, the crystal structure of YodA from *E. coli*indicates that it is a metal-binding lipocalin-like protein and it may be important in the metal stress response (David et al. 2003). In addition, the hypothetical protein ybeY belongs to the UPF0054 family and binds a metal ion. Its structure and sequence similarity to a number of predicted metal-dependent hydrolases provides a functional assignment for this protein (Zhan et al. 2005). The crystal structure of *Homo sapiens*PTD012 reveals a zinc-containing hydrolase fold (Manjasetty et al. 2006).

Proteins are a unique class of biological molecules in that they can recognize and interact with diverse substances. They contain complementary clefts and surfaces designed to bind to specific molecules. The identification of binding sites on the surface of proteins can give insights into biological function. There are many programs available for detecting and visualizing binding sites on the surface of proteins including *SURFNET*(Laskowski 1995), *CAVER*(Petrek et al. 2006) and *dxTuber*(Raunest and Kandt 2011). For example, the crystal structure of TTHB192 does not have the same signature sequence motif as the RNA recognition motif domain, however, the presence of an evolutionarily conserved basic patch on the β-sheet could be functionally relevant for nucleic acid binding (Ebihara et al. 2006). The structure for a representative of UPF0044, *E. coli*YhbY, possesses an IF3C-like core fold which is common in several RNA-binding proteins. Members of UPF0044 possess a basic surface on their β-sheet face and a proximal GKxG loop that are suggestive of an RNA recognition surface (Ostheimer et al. 2002). If the *binding site is occupied by a small molecule*carried over from the crystallization buffer or protein purification, then it is often easy to detect the functional “hot spot” in the protein structure. For example, TM841 is a 35-kDa protein comprising two separate domains with a novel fold. Therefore, it was not possible to derive any clues about this protein’s function from a comparison with proteins in the PDB. However, the electron density map clearly showed the presence of a fatty acid molecule bound in a pocket between the two protein domains, suggesting that TM841 may play a role in fatty acid transport or metabolism (Schulze-Gahmen et al. 2003). In a second example, ligands bound to the structures of p14.5, TdcF and Rv2704, which belong to the highly conserved YjgF/YER057c/UK114 protein superfamily, clearly indicated the presence of a functionally important substrate binding site. The structure of human p14.5 contains at least one benzoic acid molecule per site forming bi-dentate interactions between its carboxylate moiety and the guanidinium group of a strictly conserved arginine, Arg107 (Manjasetty et al. 2004a). Furthermore, the bonds to the ligands in the TdcF crystal structure clearly highlight the importance of the conserved residues (Burman et al. 2007). Residue conservation in an amino acid sequence or related proteins is a strong indicator of functionally important sites. Given a multiple sequence alignment of one protein against all related proteins, it is possible to determine the level of sequence conservation. Once a ‘conservation score’ for each residue in the protein has been calculated, it is useful to map these scores onto its 3D structure to see whether certain regions of the structure are more highly conserved than others. It has been demonstrated that clusters of highly conserved residues on the surface of proteins can reliably identify ligand-binding sites or protein–protein interaction sites. For example, the amino acid conservation pattern and observed metal-binding site in the crystal structure of *E. coli*YcDX indicates the location of its putative active site. All residues involved in metal coordination are invariant in the YcdX family, highlighting their functional importance (Teplyakov et al. 2003). An in-depth study is warranted to check the reliability of these ligand-binding discovery programs. The ligand or metal-binding sites observed through computational approaches can be further verified by obtaining experimental data. For example, in a recent study, a combination of experimental and bioinformatics approaches has provided a comprehensive active site analysis on the genome scale for metalloproteins, revealing new insights into their structure and function (Shi et al. 2011).

The arrangement of molecules in the asymmetric unit of the crystal and analysis of the accessible surface area buried at the subunit interfaces can be used to identify the most likely biological unit (*oligomeric state*) for a protein. The evolutionarily conserved trimeric structure of CutA1 proteins suggests a role in signal transduction (Arnesano et al. 2003). The functional annotation of the large number of structures of hypothetical proteins was published based on a bioinformatics approach (Shin et al. 2007; Yakunin et al. 2004). Therefore, crystal structures can provide insights into biological function even in the absence of any other biochemical data. However, the protein structure alone will provide conclusive functional annotation only in a limited number of cases. Therefore, SP centers collaborate with academic laboratories to resolve important questions in biology and disease. In this approach, highly organized networks of investigators apply the new paradigm of HT structure determination, which has been successfully developed at SP centers during the past decade, to study a broad range of important biological and biomedical problems. One such example is the NIH-funded *Enzyme Function Initiative*(http://enzymefunction.org).

#### Information management

SP data are hard to manage due to their complexity and the fact that different parts of the structure determination process on the same protein target are often performed at different laboratories as a collaborative effort. The file-based communications and data transactions become time consuming and sometimes unmanageable. Efficient data management techniques have been developed at SP centers to keep track of the enormous amount of data generated, which minimize duplication of effort and maximize the chances of success at each step (Haquin et al. 2008). Notable laboratory information management systems (LIMS) of SP centers include *Sesame*(Zolnai et al. 2003), *HalX*(Prilusky et al. 2005), *PiMS*(Morris et al. 2011), *SPEX Db*(Raymond et al. 2004) and *IceDB*. All the SP centers have developed their own data management systems and are linked to a centralized target registration database, *TargetDB*(Chen et al. 2004) hosted by the PDB. *PepcDB*, an extension of *TargetDB*, allows groups to report protocols and experimental details (Pan et al. 2007). Subsequently, the *Protein Structure Initiative Knowledgebase*(PSIKB) was created by integrating *TargetDB*and *PepcDB*to turn the products of the SP effort into knowledge that can be accessed by the life sciences community (Berman et al. 2009). Furthermore, a *Structural Biology Knowledgebase*(SBKB) web resource (http://www.sbkb.org) has recently been developed to aid protein research with improved features to foster collaborations between the biological community and SP centers (Gabanyi et al. 2011).

## Structural proteomics for biology

Structural knowledge of a protein clearly provides clues relating to its biological activity and physiological role. SP is one of the recent technologies that promotes drug discovery and biotechnological applications. Structural information can be used in many ways to ascertain the functional properties of cellular components. One of the crucial components for understanding the functions of novel proteins is the analysis of their experimental or modeled 3D structures. SP centers have provided an enormous impetus for methods development in structural biology and many laboratories are now actively implementing these technologies.

### Follow-on research

Scientists and engineers are now involved in utilizing structural knowledge of proteins generated by the SP approach as a basis for understanding protein function to utilize proteins in various technological applications as follow-on research. For instance, in Europe, the emphasis of the *Structural Proteomics IN Europe*(SPINE2; http://www.spine2.eu/SPINE2/index.jsp) initiative has been to apply HT technologies to systems of biological interest, the ultimate aim being to solve significant problems more effectively. Recently, *INSTRUCT*(http://www.structuralbiology.eu/) has started offering scientists access to world-class structural biology and SP infrastructures and expertise that makes such integration possible more rapidly, creating a coherent forum for structural biology. This forum will stimulate closer collaboration between scientific communities and initiatives in the life sciences. For instance, in Germany, one of the *INSTRUCT*centers, the *Protein Sample Production Facility*(http://www.pspf.de) of the Helmholtz association, provides HT *E. coli*protein expression and large-scale production technology with animal cell lines for European structural biologists. The *HT Crystallization platform*at the EMBL Grenoble Outstation, France, offers automated crystallization to European researchers. Eleven facilities from across Europe provide installations for applications including *macromolecular X*-*ray crystallography data collection*. *BioStruct*-*X*(http://www.biostructx.org/) cooperates with *INSTRUCT*aiming to provide an integrated and coordinated technology platform to all relevant methods in structural biology.

In the USA, the goal of the PSI:Biology project is to apply the paradigm of HT structure determination via highly organized networks of investigators to solve the 3D structure of proteins and macromolecular complexes representing significant biological and biomedical problems. For instance, a protein family specific platform, *GPCR*(*G*-*protein coupled receptor*) network (http://gpcr.scripps.edu/) was established to determine the high-resolution structure and function of GPCRs distributed broadly across the phylogenetic family tree. GPCRs mediate many important cellular signal transduction events related to differentiation, proliferation, angiogenesis, cancer, development and cell survival. GPCRs represent the targets for 60–70% of drugs currently in development. The recent progress and success in the structure determination of GPCRs by utilizing SP technologies like LCP (Lipidic Crystal Phase) crystallization (Cherezov 2011) and micro-crystallography technologies for data collection (10 × 10 μm beam size, microfocus beamline, Pilatus 6 M detector) have been described (Cherezov et al. 2009; Shimamura et al. 2011). In addition, HT-enabled structural biology partnerships have also been established. For example, the *IFN*(Immune Function Network), a consortium of immunologists, geneticists, computational biochemists and HT structural biologists, is committed to the coordinated structural, in vitro biochemical and in vivo functional analyses of secreted molecules and ectodomains of cell surface molecules which control adaptive and innate immunity (Chattopadhyay et al. 2009). The research activities of the IFN will be conducted in collaboration with the NYSGRC (http://www.nysgxrc.org/). The *TB Structural Genomics Consortium*is a worldwide consortium of scientists developing a foundation for tuberculosis diagnosis and treatment by determining the 3D structures of proteins from *Mycobacterium tuberculosis*(Mtb). The consortium seeks to solve structures of proteins that are of great interest to the TB biology community (http://www.webtb.org/) (Musa et al. 2009). Tuberculosis poses a global health emergency, which has been compounded by the emergence of drug-resistant Mtb strains. For instance, the protein structure of isocitrate lyase, a persistence factor of Mtb (Sharma et al. 2000), has been extensively studied and advances in technology have enabled the assembly of HT pipelines that can be used for the development of glyoxylate cycle inhibitors as new drugs for the treatment of this disease (Munoz-Elias and McKinney 2005). Further, as a step toward the better integration of protein 3D structural information in cancer systems biology, NESG has constructed a *HCPIN*(*Human Cancer Pathway Protein Interaction Network*) by analzsing several classical cancer-associated signaling pathways and their physical protein–protein interactions (Huang et al. 2008).

### Systems biology and biotechnology

In systems biology, proteins are visualized as a network of interconnected dots. To understand the complexity of cellular function, one should know the detailed 3D behaviors of all the available dots which form the basis of life. Furthermore, the structures of these proteins could provide quantitative parameters to help elucidate functional networks through knowledge of protein function, evolution and interactions. The protein structures generated by SP can be used for the assignment of domain structure, functional annotation and the prediction of interaction partners in biochemical pathways (Harrill and Rusyn 2008). The structural information can be used to further characterize large-scale protein interaction networks by providing the key functional properties of cellular components (Beltrao et al. 2007).

Biotechnology embraces the bioproduction of fuels and chemicals from renewable sources. Sustainable energy is a major problem in the twenty-first century. If biofuels are to be part of the solution, this field must accept a degree of scrutiny unprecedented in the development of a new industry. That is because sustainability deals explicitly with the role of biofuels in insuring the well-being of our planet, our economy and our society, both today and in the future. The development of detailed kinetic models that include accurate regulatory network parameters will facilitate the identification of enzymatic bottlenecks in the metabolic pathways that could be harnessed to achieve biofuels overproduction. The latest advances in SP will continue to identify the biocatalysts, which power the development of enzyme reactors for producing substantial amounts of biofuels (Daniels et al. 2011). Some biomolecules are robust enough to be used in biotechnological applications. For instance, enzymes can be used to break down starch to form sweeteners. The structure–function relationship of *E. coli*arabinose isomerase, ECAI, advanced its application in tagatose (a new sweetener) production (Manjasetty et al. 2010; Manjasetty and Chance 2006) (Fig. 7a).

Nanobiotechnology is a novel branch of futuristic science and engineering. A nanobiomachine is a machine formed by a biomolecule with a nanoscale diameter. The knowledge of a protein sequence provides the basis for understanding these nanobiomachines, which ultimately describe its functional significance. The structural and functional knowledge of a protein is essential to utilize proteins in nanotechnology applications and to develop bionanodevices. Glucose oxidase is a small, stable enzyme that oxidizes glucose into glucolactone, converting oxygen into hydrogen peroxide in the process. It is used as the heart of *biosensors*that measure the amount of glucose in the blood. Insights from protein structure can be crucial in engineering proteins for nanotechnology applications.

### Models of protein structures and drug design

SP projects around the globe were established to determine the structures of proteins in an HT, automated fashion. However, despite the advances made by SP organizations in terms of automation, throughput and methodology development, the structures of certain classes of proteins, such as membrane proteins, are still notoriously difficult to determine. This warrants alternative techniques to generate models for these proteins to enhance our understanding of their physical and chemical properties. This has led to the development of a large number of bioinformatics tools capable of generating models for these novel proteins. Among them, *MODELLER*(Eswar et al. 2008) and *ROSETTA*(Das and Baker 2008) represent two of the best protein structure prediction servers. *MODELLER*generates a model of an unknown protein using a template structure generated by the SP approach, whereas *ROSETTA*provides ab initio structure prediction of the unknown protein. Biomodeling provides the ability to understand the physicochemical properties of proteins of biomedical importance with undetermined 3D structures. It involves a range of computerized techniques based on quantum physics and experimental proteomics data to predict and correlate biological properties at the molecular level. Statistical and regression analysis techniques are the best methodologies and are capable of predicting geometries, energies, and electronic and spectroscopic properties. Homology-based modeling, as applied by *MODELLER*and *ROSETTA*, relies on sequence alignments between proteins of known structure and the target protein. The accuracy of the calculated model is dependent on the accuracy of the sequence alignment and the divergence between target and template. The most accurate alignments are obtained by iterative and profile or *HMM*(*Hidden Markov Models*)-based methods. In addition, structural data can be used to verify and improve alignments.

Biomodeling has become a valuable and essential tool in the drug design and discovery process. Drug design is a 3D puzzle where small drug molecules are fitted into the active site of a protein. The factors which affect protein–ligand interactions can be characterized by molecular docking and studying quantitative structure activity relationships (*QSAR*). Traditionally, drug discovery relies on a stepwise synthesis and screening of large numbers of compounds to optimize drug activity profiles. The design of new and more potent drugs against diseases such as cancer, AIDS and arthritis can be aided using bioinformatics tools such as computer-assisted drug design (*CADD*) or computer-assisted molecular design (*CAMD*). Structural bioinformatics tools not only have the potential to build predictive models of the proteins of biomedical interest, but also help to bring new drugs to market. Complementary *in silico*methods, such as structure-based drug design (*SBDD*), incorporate the knowledge from high-resolution 3D protein structures generated by SP to probe structure–function relationships, identify and select therapeutically relevant targets (assessing druggability), study the molecular basis of protein–ligand interactions, characterize binding pockets, develop target-focused compound libraries, identify hits by HT docking (*HTD*) and optimize lead compounds, all of which can be used to rationalize and increase the speed and cost-effectiveness of the drug discovery process. An analysis of the results obtained by several docking and modeling programs has shown that, in most cases, they can work well. Most of the programs used in drug discovery have incorporated subroutines to identify false positives or negatives using scoring functions, which has led to a significant improvement in hit rates.

## Conclusion

Contributions from SP are mainly twofold: first, novel structural information has been generated to understand the proteome of various organisms; second, innovative HT technologies have been developed for protein structure determination. These technologies and related structural information have, in turn, been exploited by biologists in many different ways in broad areas of life sciences research. However, structural knowledge alone is not sufficient to fully understand a protein’s cellular role. Hence, a major bottleneck is studying a protein’s behavior and dynamics within larger macromolecular assemblies and protein–protein interactions within a cellular pathway. Research such as this will drive the application of SP in the decades that follow.
